# Recommended Vaccinations During Adolescence: Parents’ Knowledge and Behaviors

**DOI:** 10.3390/vaccines12121342

**Published:** 2024-11-28

**Authors:** Giovanna Paduano, Vincenza Sansone, Concetta Paola Pelullo, Silvia Angelillo, Francesca Gallè, Gabriella Di Giuseppe

**Affiliations:** 1 Department of Experimental Medicine, University of Campania “Luigi Vanvitelli”, 80138 Naples, Italy; giovanna.paduano@unicampania.it (G.P.); vincenza.sansone@unicampania.it (V.S.); silvia.angelillo@studenti.unicampania.it (S.A.); gabriella.digiuseppe@unicampania.it (G.D.G.); 2 Department of Medical, Human Movement and Well-Being Sciences, University of Naples “Parthenope”, 80133 Naples, Italy; francesca.galle@uniparthenope.it

**Keywords:** adolescents, behaviors, knowledge, parents, Italy, survey, vaccination

## Abstract

Background: This study aims to evaluate parents’ knowledge about vaccination targeted for adolescents. Methods: The cross-sectional survey was conducted between February and April 2024 in a sample of parents of adolescents attending middle and high schools in Southern Italy. Results: Only 10.9% of parents correctly answered all the questions related to the knowledge of vaccinations during adolescence. The results of the multivariate logistic regression model showed that male parents, those who were married/cohabitants, those who had a university degree/masters/PhD, those who were unemployed, those who had a higher number of cohabitants, those who had more than one son/daughter, and parents of older adolescents were significantly more likely to have correct knowledge regarding the vaccinations during adolescence. Overall, more than half of the parents reported that their sons/daughters received the vaccinations indicated during adolescence. Moreover, regarding the vaccinations recommended during adolescence, only 37.2% of parents reported having vaccinated their sons/daughtesr with HPV and tetravalent meningococcal vaccinations. The results of the multivariate logistic regression model showed that those who had correct knowledge regarding vaccination during adolescence, those who had only one son/daughter, parents of older adolescents, those who had a daughter, those who had at least one chronic disease, and those who had a higher number of cohabitants were significantly more likely to have vaccinated their sosn/daughters with HPV and tetravalent meningococcal vaccinations. Conclusions: These findings underlined the need to plan immunization campaigns for adolescents and their parents, with the implementation of educational programs specifically targeted to these groups.

## 1. Introduction

Until the 2000s, vaccinations seemed to be the prerogative of infant or children of preschool age, since the national vaccination calendar did not provide vaccines to be administered to adolescents other than the booster dose of the diphtheria, tetanus, acellular pertussis and polio vaccine (dTaP-IPV) [1,2]. Over the years and with the progress of scientific research, some vaccines have also been included in the vaccination schedule for adolescents [3,4,5] for the primary prevention of infectious diseases and cancers [6,7,8], such as meningococcal C (MenC) or tetravalent meningococcal (MenACWY), one dose of the meningococcal B, and Human Papillomavirus (HPV) vaccine. This has raised greater attention on adolescence, which has become crucial for vaccination campaigns aimed at protecting the individual and other age groups [9,10,11]. Indeed, the adolescent has frequent moments of socialization in schools, gyms, and meeting places, which favor the spread of infections; moreover, since the onset of sexual activity often occurs during adolescence, this age group represents the primary target for the prevention of cervical cancer and other HPV-related diseases [12]. According to the Italian National Prevention Vaccination Plan (PNPV) recommendation, adolescents should receive at the age of 11–12 years two or three doses of HPV vaccine and at 12–18 years the first booster dose dTaP-IPV [1]. Although the target coverage is ≥95%, the latest data from the Ministry of Health report that coverage for the meningococcal and HPV vaccines is far from being achieved [13,14], despite the coverage for mandatory ones that is more satisfying [15]. Unfortunately, it must be considered that over the years, especially for some vaccines, due to the spread of false, alarming messages regarding possible harmful effects of vaccinations and the COVID-19 pandemic, vaccine hesitancy has increased and, therefore, it has not been possible to reach the threshold of ≥95% coverage of vaccinated subjects [1,16]. This situation could determine that some vaccine-preventable diseases, typically occurring during childhood, have moved towards adolescence and adulthood, with more serious symptoms. Indeed, to prevent infections in adolescents, we can act prior to natural exposure when vaccinations are more effective [17]. Studies have also demonstrated that earlier administration of the vaccine results in greater immunogenicity and longer-lasting protection [18,19].

One of the major difficulties that all health professionals must deal with is the promotion of vaccinations in adolescence [20,21]. Adolescent immunizations are important to prevent serious and lethal illnesses, and even cancer in adulthood. Schools can act as catalysts for family communication regarding health services and promoting preventive behaviors. Establishing a dialogue between adolescents and their parents is very complex because, at this age, adolescents assert their desire for autonomy and ask to participate in decisions, but at the same time they do not always worry about their health and, therefore, are not very sensitive to prevention, despite being at an age that exposes them to various risks of infection [22]. Moreover, decisions on their health are legally taken by parents who need to be informed in order to promote prevention.

In the literature, several studies highlight the main barriers to vaccination adherence in adolescence, such as poor knowledge among parents about the risks of some infectious diseases [23,24,25], vaccine hesitancy [26,27,28], inadequate information, especially from physicians, in whom adolescents and parents usually place their trust, in addition to a scarce perception of the importance of vaccinations and excess of concern about their safety. Therefore, considering the choice to get vaccinated as the result of a complex interaction between parents and adolescents, the aim of this study is to evaluate parents’ knowledge about vaccinations targeted for this age group.

## 2. Materials and Methods

### 2.1. Setting and Participants

This cross-sectional survey was part of a larger study of adolescents’ and parents’ lifestyles and healthy behaviors. Data were collected between February and April 2024 from a sample of parents of adolescents attending middle and high school in Naples and Avellino, two cities located in Campania region, Southern Italy. From the list of the 78 public schools in this area, four middle and five high schools were casually chosen and, in each school, classes were randomly selected to be involved in the study. Adolescents aged from 11 to 19 years were included in the survey. Those attending middle school but aged less than 11 years were excluded, since in the Italian National Vaccination Calendar there is no indication of vaccination for them. The minimum sample size was designed before the beginning of the study, assuming a 95% confidence interval, an alpha error of 5%, a response rate of 50%, and, since previous studies presented divergent results, an expected 50% adhering to the recommended vaccination campaign for adolescents [29,30].

Therefore, the minimum sample size was estimated to be 385 parents. The study protocol and the questionnaire were approved by the Ethics Committee of the University of Campania “Luigi Vanvitelli” (prot. N.0018199/i/2024).

### 2.2. Data Collection

To involve the selected schools in the data collection, the heads were approached to plan an introductory meeting, during which a member of the research team explained the study aims and delivered a letter defining the procedures for data collection. After the heads consented, part of the research team went to the selected class to explain the study procedure. Each adolescent was handed an envelope containing a cover letter, an informed consent form, and the questionnaire. The cover letter described the purpose of the study and gave information on how to participate, how anonymity and confidentiality of the data would be guaranteed, and that no payment or contributions would be given to those involved in the study. The adolescents were requested to hand over the envelope to one of their parents and to store the fulfilled questionnaire closed in the envelope, whereas the signed informed consent form had to be stored separately. According to the school’s head, a team member returned ten days later to collect the filled questionnaires and the informed consent forms.

### 2.3. Questionnaire

To collect information about adolescents’ health, knowledge and behaviors related to vaccinations during adolescence, a questionnaire based on the studies conducted by some of the authors was used [31,32,33]. The questionnaire consisted of four sections. The first collected socio-demographic and anamnestic characteristics of parents and adolescents, such as age, gender, education level, occupational status, number of cohabitants, number of sons/daughters, and the presence of chronic diseases. The second section investigated participants’ knowledge and behaviors regarding recommended vaccinations during adolescence. It included 7 true or false questions exploring knowledge (the booster for the dTaPvaccination should be taken at 12 years; the booster for the IPV vaccination should be taken at 12 years; the booster for the dTaP vaccination is mandatory and free of charge; the booster for the IPVvaccination is mandatory and free of charge; HPV vaccination is also recommended in males; HPV vaccination is free of charge for males and females; and MenACWY vaccination is free of charge and recommended for ages 12 to 18; those who answered correctly to all the questions were considered as having correct knowledge. One question asked whether the adolescent had undergone the following vaccinations: anti-dTaP-IPV, anti-HPV, and anti- MenACWY, with three options (“yes”, “no”, and “do not remember”) for each vaccination. Parents were asked to indicate their reasons for having or not having vaccinated their sons/daughters from a list of twelve options, with the possibility to select multiple answers. The third section examined the sources of information among multiple proposed answers and the need for additional information on adolescents’ health.

The questionnaire was tested with a pilot study among 100 parents of adolescents to confirm clarity and feasibility. Since no changes were made, the answers were included in the final sample.

### 2.4. Statistical Analysis

Descriptive statistics were conducted to present the main results, including frequencies and proportions for categorical variables. Continuous data were described as means and standard deviations when normally distributed.

The outcomes of interest were the following: having correct knowledge regarding the vaccinations during adolescence (Model 1) (0 = no; 1 = yes) and having vaccinated their son/daughter with the HPV and MenACWY vaccinations during adolescence (0 = no; 1 = yes) (Model 2). The following independent variables were tested in both models: parent’s age (continuous), parent’s gender (male = 0; female = 1), marital status (unmarried/separated/divorced/widowed = 0; married/cohabitants = 1), having an occupation (no = 0; yes = 1), having university degree/masters/PhD (no = 0; yes = 1), having at least one chronic disease (no = 0; yes = 1), having someone with at least one chronic disease in the family (no = 0; yes = 1), number of cohabitants (continuous), number of sons/daughters (1 = 0; ≥2 = 1), adolescent’s age (continuous), adolescent’s gender (male = 0; female = 1), sources of information (none = 1; physicians = 2; others (media/journals/Internet/social/school/job/friends/relatives) = 3), and the need for additional information about adolescents’ health (no = 0; yes = 1). Having correct knowledge regarding vaccinations during adolescence (0 = no; 1 = yes) was added in Model 2. The strength and direction of associations between the independent variables and outcomes of interest were estimated in the multivariate regression models with odds ratios (ORs) and 95% confidence intervals (CIs). All analyses were two-sided, and a *p*-value of 0.05 or less was set as statistically significant. The statistical analysis was performed with Stata software, version 17 [34].

## 3. Results

### 3.1. Socio-Demographic and Anamnestic Characteristics of Parents and Adolescents

In total, 1111 questionnaires were sent out and 727 parents agreed to participate in the study, with a response rate of 65.4%. A total of 11 parents were excluded because the age of their sons/daughters was 10 years. Table 1 summarizes the socio-demographic and anamnestic characteristics of parents and adolescents. A large majority of responding parents were females (78.3%) and married/cohabitants with a partner (88.9%), and the average age was 47.3 years (range: 28–78 years). In less than half of the families (48.8%), there was at least one parent with a university degree/masters/PhD, and 78.9% were employed. With regard to anamnestic characteristics, only 17.3% had at least one chronic disease, and 14.6% had someone with at least one chronic disease in the family. The average number of cohabitants was 2.9 (range: 1–7), and most of the respondents (78.2%) had two or more sons/daughters, with an average age of 12.6 years (range: 11–19 years), and 44.8% of adolescents were females.

### 3.2. Parents’ Knowledge Regarding Vaccination During Adolescence

Table 2 presents the answers to the questions on parents’ knowledge regarding vaccinations during adolescence. Regarding the knowledge on anti-dTaP-IPV vaccinations, almost all respondents, respectively, 85.1% and 78.5%, correctly reported that the booster is mandatory and free of charge. Only 37.7% and 30.3% correctly reported that the anti-dTaP-IPV vaccine boosters should be taken at 12 years, respectively. Moreover, regarding the recommended vaccinations during adolescence, anti-HPV and anti-MenACWY vaccinations, the majority of the parents correctly reported that the HPV vaccination is also recommended for males (89.8%) and that this vaccination is free of charge for males and females (85.1%). With regard to MenACWYvaccination, more than half of parents (54.7%) correctly reported that the vaccination is free of charge and recommended for ages 12 to 18. Indeed, 10.9% of parents correctly answered all the questions related to knowledge on vaccinations during adolescence.

The results of the multivariate logistic regression model showed that the following factors were significantly associated with correct knowledge regarding the vaccinations during adolescence: being married/cohabitants (OR = 5.62; 95% CI = 1.11–28.72), having a university degree/masters/PhD (OR = 2.47; 95% CI = 1.24–4.92), having more than one son/daughter (OR = 3.77; 95% CI = 1.27–11.23), and parents of older adolescents (OR = 1.22; 95% CI = 1.04–1.44). Moreover, male parents (OR = 0.39; 95% CI = 0.19–0.81), those who were unemployed (OR = 0.43; 95% CI = 0.19–0.95), and those who had a higher number of cohabitants (OR = 0.48; 95% CI = 0.25–0.96) were significantly less likely to have correct knowledge regarding the vaccinations during adolescence (Model 1 in Table 3).

### 3.3. Parents’ Behaviors Towards Vaccination During Adolescence

The details of the parents’ behaviors towards vaccination during adolescence are shown in Table 4. Overall, more than half of the parents reported that their sons/daughters received the vaccinations indicated during adolescence: diphtheria (54.3%), tetanus (63%), pertussis (64.4%), polio (54%), and MenACWY (60.6%). Only for the HPV vaccination, less than half of the parents (48.6%) reported that their sons/daughters received the vaccination. Among parents who declared that their sons/daughters received the vaccinations, the most commonly reported reasons were having received a recommendation from the primary care pediatrician or physician (53.3%), considering vaccinations effective (50.8%), being favorable to vaccinations (47.6%), and considering vaccinations safe (32.3%). Major reasons for refusing vaccinations were a lack of vaccine recommendations from the primary care pediatrician or physician (34.7%), considering the vaccinations received in the first years of life sufficient (19.6%), and a fear of adverse events (16.9%).

Regarding the vaccinations recommended during adolescence, HPV and MenACWY vaccinations, only 37.2% of parents reported having vaccinated their sons/daughtesr with these vaccinations.

The results of the multivariate logistic regression model showed that the following factors were significantly associated with having vaccinated their sons/daughters with the HPV and MenACWYvaccinations during adolescence: having correct knowledge regarding vaccinations during adolescence (OR = 4.14; 95% CI = 2.23–7.69), parents of older adolescents (OR = 1.23; 95% CI = 1.09–1.38), having a daughter (OR = 1.91; 95% CI = 1.29–2.82), having at least one chronic disease (OR = 1.78; 95% CI = 1.08–2.96), and having a high number of cohabitants (OR = 1.54; 95% CI = 1.14–2.08). Moreover, those who had only one son/daughter (OR = 0.41; 95% CI = 0.22–0.73) were significantly less likely to have vaccinated their sons/daughters with the HPV and MenACWYvaccinations during adolescence (Model 2 in Table 3).

### 3.4. Sources of Information

Almost all respondents (93.3%) had received information about adolescents’ health and the most common sources of information were physicians (67.7%). Finally, 52.4% of parents acknowledged that they needed additional information about adolescents’ health.

## 4. Discussion

The results of this study showed that parents’ awareness of vaccinations targeted for adolescents was generally good in the examined sample, and participants reported physicians as the main source of information on this topic. This is in line with other investigations and accounts for lower vaccine hesitancy in the populations examined [35,36,37]. However, besides the availability and the offer of these vaccines, most of the participants did not know the right age for their administration, which highlights the need for further information, which was also declared by about the half of the sample.

Better knowledge was found to be related to parents’ sex, marital status, and education level, and to the number and ages of their children. The result concerning fathers’ knowledge is not consistent with previous studies [38,39]. However, it should be noted that these investigations were carried out in different countries and involved only fathers, which did not allow us to make a gender comparison. Instead, our finding is in line with that of Alghamdi et al., who reported the same effect of gender on beliefs and knowledge about vaccines [40]. The correspondence between the parental educational level and vaccine knowledge and attitudes has been reported in previous studies performed in Italy and other countries [40,41,42,43]. As for the relationship with the number of children, even the study by Alghamdi et al. reported higher rates of correct beliefs and knowledge among parents with only one child [40], while that of Ashkenazi et al. showed lower knowledge about the measles vaccination in single-child parents [44]. The same study also reported lower vaccine knowledge among single parents with respect to the married ones, confirming our result for this aspect [44]. With regard to the adherence to vaccination campaigns, although the majority of the sample declared that their children underwent the available vaccinations, less than the half of them reported HPV vaccination. However, the last data available for HPV vaccination coverage in Italy, which refer to the year 2022, report wide ranges across birth cohorts (1.73–14.20% for adolescent males and 23.96–52.45% for adolescent females) from the Campania region [13]. In this scenario, it is possible to assume that our data stand for an increasing coverage in the target population.

Adherence to recommended vaccinations for adolescents seems to be related to having a chronic disease and living with a higher number of cohabitants, but also to the sex, number and age of children. As for the first model, it can be argued that suffering from a chronic illness plays a role in increasing attention toward health protection [45]. The association between compliance with vaccinations and children’s gender may be attributed to the delayed introduction of the HPV vaccine offer to males in Italy, which has determined lower rates of vaccination coverage in this gender throughout the Italian territory [13]. As for the number of children, the study by Ashkenazi et al., in accordance with our findings, showed higher vaccine hesitancy among parents with only one child [44]. Furthermore, adherence to vaccination programs was found to be positively related to knowledge about vaccinations, which is in agreement with other studies [43]. The association between parents’ knowledge and attitudes towards vaccinations and children’s age is interesting and deserves further investigations. This study has some limitation. First of all, the sample was composed of parents who voluntarily chose to participate in the study after having known the topic. Therefore, it is possible that a higher number of parents sensitive to health topics adhered to the investigation, and this could have affected the results. Furthermore, data were self-reported, and they could have provided inaccurate information. However, this study allowed us to assess the level of awareness and the adhesion to vaccinations directly among parents, who are responsible for the health of their children. Considering that updated vaccination rates are not available yet for the Italian population, the picture emerging from our study can provide some indication about the effectiveness of vaccine campaigns. Finally, COVID-19 and flu vaccine coverage was not explored in this survey. In the post-pandemic period, the aspect related to parents’ knowledge and behaviors toward COVID-19 vaccines is a potential driver of their relationship with other vaccinations, especially because the pandemic delayed or reduced vaccine uptake.

## 5. Conclusions

In conclusion, the findings of this study allow us to highlight some aspects that need to be addressed in order to improve infectious disease control through vaccinations. In particular, it seems that some parent categories, such as mothers, single parents, those with a lower educational level, and those who have more than one child, show higher information needs. When planning immunization campaigns for adolescents and their parents, the implementation of educational programs specifically targeted to these groups should be considered.

## Figures and Tables

**Table 1:** Main characteristics of parents participating in the survey (n: 716).

Characteristics	Total (n: 716)	
Socio-Demographic	N	%
Parent’s gender (711) *		
Male	148	20.8
Female	595	78.3
Parent’s age in years (701) *	47.3 ± 5.6 ^a^	
Marital status (705) *		
Unmarried/widowed/divorced	78	11.1
Married/cohabitants	627	88.9
Education level (696) *		
High school	361	51.2
University degree/masters/PhD	344	48.8
Partner’s education level (616) *^°^		
High school	401	65.1
University degree/masters/PhD	215	34.9
Occupation (696) *		
Unemployed	147	21.1
Employed	549	78.9
Partner’s occupation (606) *^°^		
Unemployed	44	7.3
Employed	562	92.7
Having at least one chronic disease (701) *		
No	580	82.7
Yes	121	17.3
Having someone with at least one chronic disease in the family (680) *		
No	581	85.4
Yes	99	14.6
Number of cohabitants (703) *	2.9 ± 0.9 ^a^	
Number of sons/daughters (712) *		
1	155	21.8
≥2	557	78.2
Adolescent’s age in years, (continuous) (699) *	12.6 ± 1.7 ^a^	
Adolescent’s gender (709) *		
Male	391	55.2
Female	318	44.8
**Use of sources of information**		
None	47	6.7
Physicians	472	67.7
Others (media/journals/Internet/social/school/job/friends/relatives)	178	25.5
Need to receive additional information about adolescents’ health (695) *		
No	331	47.6
Yes	364	52.4

^a^ Means + standard deviations. * The numbers for each item may not add up to total number of study population due to missing values. ^°^ Only among those who had a partner.

**Table 2:** Parents’ knowledge regarding adolescents’ vaccinations (n: 716).

Knowledge	N	%
The booster for the anti-diphtheria/tetanus/pertussis vaccination should be taken at 12 years (655) *		
Incorrect	408	62.3
Correct	247	37.7
The booster for the anti-polio vaccination should be taken at 12 years (630) *		
Incorrect	439	69.7
Correct	191	30.3
The booster for the anti-diphtheria/tetanus/pertussis vaccination is mandatory and free of charge (646) *		
Incorrect	96	14.9
Correct	550	85.1
The booster for the anti-polio vaccination is mandatory and free of charge (629) *		
Incorrect	135	21.5
Correct	494	78.5
Human Papillomavirus vaccination is also recommended for males (666) *		
Incorrect	68	10.2
Correct	598	89.8
Human Papillomavirus vaccination is free of charge for males and females (659) *		
Incorrect	98	14.9
Correct	561	85.1
Tetravalent meningococcal vaccination is free of charge and recommended for ages 12 to 18 (642) *		
Incorrect	291	45.3
Correct	351	54.7

* The number for each item may not add up to total number of study population due to missing values.

**Table 3:** Multivariate logistic regression models to identify those factors predicting the above-mentioned outcomes of interest.

* Model 1. Parent’s Correct Knowledge Regarding Vaccinations During Adolescence			
**Log Likelihood = −175.74, χ^2^ = 31.26 (11 df), *p* = 0.0010**			
**Variable**	**OR**	**95% CI**	***p***
**Parent’s gender**			
Male	1 ^a^		
Female	0.39	0.19–0.81	0.011
**Marital status**			
Unmarried/widowed/divorced	1 ^a^		
Married/cohabitants	5.62	1.11–28.72	0.038
**Education level**			
High school	1 ^a^		
University degree/masters/PhD	2.47	1.24–4.92	0.010
**Occupation**			
Unemployed	1 ^a^		
Employed	0.43	0.19–0.95	0.037
**Number of cohabitants**	0.48	0.25–0.94	0.031
**Number of sons/daughters**			
1	1 ^a^		
≥2	3.77	1.27–11.23	0.017
**Adolescent’s age in years, (continuous)**	1.22	1.04–1.44	0.015
**Parents’ age in years, (continuous)**	0.95	0.91–1.01	0.072
**Having at least one chronic disease**			
No	1 ^a^		
Yes	1.77	0.87–3.57	0.113
**Having someone with at least one chronic disease in the family**			
No	1 ^a^		
Yes	0.59	0.23–1.48	0.261
**Use of sources of information**			
None	0.46	0.11–2.04	0.308
Physicians	1 ^a^		
Others (media/journals/Internet/social/school/job/friends/relatives)	Backward elimination		
**^°^ Model 2. Having Vaccinated Their Sons/Daughters with the HPV and MenACWY Vaccinations During Adolescence**			
**Log Likelihood = −326.48, χ^2^ = 59.85 (9 df), *p* > 0.001**			
**Variable**	**OR**	**95% CI**	***p***
**Parent’s gender**			
Male	1 ^a^		
Female	0.73	0.45–1.21	0.220
**Parents’ age in years, (continuous)**	0.98	0.95–1.02	0.379
**Number of sons/daughters**			
1	1 ^a^		
≥2	0.41	0.22–0.73	0.003
**Adolescent’s age in years, (continuos)**	1.23	1.09–1.38	<0.001
**Adolescent’s gender**			
Male	1 ^a^		
Female	1.91	1.29–2.82	0.001
**Number of cohabitants**	1.54	1.14–2.08	0.005
**Having at least one chronic disease**			
No	1 ^a^		
Yes	1.78	1.08–2.96	0.025
**Parent’s correct knowledge regarding adolescents’ vaccinations**			
No	1 ^a^		
Yes	4.14	2.23–7.69	<0.001
**Marital status**			
Unmarried/widowed/divorced	1 ^a^		
Married/cohabitants	0.66	0.31–1.14	0.289
**Having someone with at least one chronic disease in the family**			
No	1 ^a^		
Yes	0.61	0.35–1.08	0.089
**Use of sources of information**			
None	Backward elimination		
Physicians	1 ^a^		
Others (media/journals/nternet/social/school/job/friends/relatives)	1.29	0.84–1.97	0.244

^a^ Reference category. * The following variables were deleted by the backward elimination procedure: adolescent’s gender and need to receive additional information about adolescents’ health. **^°^** The following variables were deleted by the backward elimination procedure: parent’s gender, parent’s age in years, education level, occupation, and need to receive additional information about adolescents’ health.

**Table 4:** Parents’ behaviors towards adolescents’ vaccinations.

Vaccinations	N	%
Diphtheria (501) *^a^		
No/Don’t remember	229	45.7
Yes	272	54.3
Tetanus (505) *^a^		
No/Don’t remember	187	37
Yes	318	63
Pertussis (504) *^a^		
No/Don’t remember	180	35.6
Yes	324	64.4
Polio (499) *^a^		
No/Don’t remember	230	46
Yes	269	54
Human Papillomavirus (510) *		
No/Don’t remember	350	51.4
Yes	331	48.6
Tetravalent meningococcal (509) *^a^		
No/Don’t remember	269	39.4
Yes	414	60.6

* The number for each item may not add up to total number of study population due to missing values. **^a^** Only among those who had a children aged ≥ 12 years.

## Data Availability

The data presented in this study are available upon request from the corresponding author.
